# Expert Coaching in Weight Loss: Retrospective Analysis

**DOI:** 10.2196/jmir.9738

**Published:** 2018-03-13

**Authors:** Stefanie Lynn Painter, Rezwan Ahmed, Robert F Kushner, James O Hill, Richard Lindquist, Scott Brunning, Amy Margulies

**Affiliations:** ^1^ Retrofit, Inc Chicago, IL United States

**Keywords:** body mass index, coaching, feedback, obesity, overweight, weight loss, weight reduction program

## Abstract

**Background:**

Providing coaches as part of a weight management program is a common practice to increase participant engagement and weight loss success. Understanding coach and participant interactions and how these interactions impact weight loss success needs to be further explored for coaching best practices.

**Objective:**

The purpose of this study was to analyze the coach and participant interaction in a 6-month weight loss intervention administered by Retrofit, a personalized weight management and Web-based disease prevention solution. The study specifically examined the association between different methods of coach-participant interaction and weight loss and tried to understand the level of coaching impact on weight loss outcome.

**Methods:**

A retrospective analysis was performed using 1432 participants enrolled from 2011 to 2016 in the Retrofit weight loss program. Participants were males and females aged 18 years or older with a baseline body mass index of ≥25 kg/m², who also provided at least one weight measurement beyond baseline. First, a detailed analysis of different coach-participant interaction was performed using both intent-to-treat and completer populations. Next, a multiple regression analysis was performed using all measures associated with coach-participant interactions involving expert coaching sessions, live weekly expert-led Web-based classes, and electronic messaging and feedback. Finally, 3 significant predictors (*P*<.001) were analyzed in depth to reveal the impact on weight loss outcome.

**Results:**

Participants in the Retrofit weight loss program lost a mean 5.14% (SE 0.14) of their baseline weight, with 44% (SE 0.01) of participants losing at least 5% of their baseline weight. Multiple regression model (*R*^2^=.158, *P*<.001) identified the following top 3 measures as significant predictors of weight loss at 6 months: expert coaching session attendance (*P*<.001), live weekly Web-based class attendance (*P*<.001), and food log feedback days per week (*P*<.001). Attending 80% of expert coaching sessions, attending 60% of live weekly Web-based classes, and receiving a minimum of 1 food log feedback day per week were associated with clinically significant weight loss.

**Conclusions:**

Participant’s one-on-one expert coaching session attendance, live weekly expert-led interactive Web-based class attendance, and the number of food log feedback days per week from expert coach were significant predictors of weight loss in a 6-month intervention.

## Introduction

Worldwide, 1.9 billion adults are classified as being overweight or obese with the United States leading the globe [1,2]. This preventable disease is considered the driver of rising health care costs, and the annual direct and indirect health care costs have risen to $1.42 trillion [2].

In 2014, the direct medical costs of health conditions caused by overweight and obesity amounted to US $427.8 billion [2]. Indirect costs, such as absenteeism or loss of productivity due to disease, totaled US $988.8 billion [2]. With 70.7% of US adults being overweight or obese, employers spend an additional US $4000 more per year on an employee with obesity than on a healthy weight employee through costs related to health care, productivity, and job absenteeism [3-5]. According to the 2017 Employer Health Benefits Survey, 85% of employers provide health and wellness programs to prevent and manage chronic diseases [6]. Employer-sponsored weight management programs come in a variety of packages, including self-guided, group coaching, and individualized coaching related to activity, nutrition, and behavior change [7-11].

Weight management programs offering coaches to support participants have been shown to be more effective in participant engagement and weight loss success [7-9]. Females are more successful with weight loss programs that include direct and protocol-driven coaching around diet, physical activity, and engagement, whereas males tend to underuse coaches [12,13]. However, both males and females do benefit from coaches to increase engagement and weight loss success [9,12,13].

Offering education around behavior change and accountability for adherence of implementing information learned is one benefit of providing coaches with weight management programs. Face-to-face coaching sessions with weekly email contact from a coach was successful in helping participants lose at least 10% of initial body weight [14]. Alternatively, offering weekly email behavior coaching and monthly individualized coaching telephone calls has also shown to improve adherence to health-related strategies, decrease health risk factors, and improve weight loss [15-17]. In addition to individualized coaching, weekly behavioral change lessons, weekly individualized self-monitoring feedback, and an Web-based community group have also been shown to increase likelihood of achieving 5% weight loss in 6 months, 10% weight loss in 12 months, and maintenance of weight loss over 2 years [11,18,19].

Self-monitoring is important in achieving greater weight loss [20]. Coach-provided individualized feedback around self-monitoring increases consistency in both men and women [20]. Personalization proves to be more effective than automated emails providing general health information or tips specifically around nutrition and behavior [21-24].

The purpose of this study was to analyze the participant and coach interaction in a 6-month weight loss intervention administered by Retrofit (see Multimedia Appendix 1), a personalized weight management and Web-based disease prevention solution. The interactions were evaluated for their association with weight loss to determine the level of impact on predicting weight loss outcomes. Additionally, each type of interaction was evaluated independently to assess the association between the interaction and weight loss to determine best practices for expert coaches.

## Methods

### Study Design

A retrospective analysis was performed to assess the impact of expert coaching during a 6-month weight loss intervention using deidentified data from the Retrofit weight loss program. Various measures were designed to quantify coach-participant interactions involving one-on-one expert coaching sessions, live weekly expert-led interactive Web-based classes, food and exercise log feedback, and electronic messages. All measures were included in a multiple regression analysis to predict weight loss during the intervention. Finally, 3 statistically significant (*P*<.001) expert coaching measures were analyzed in depth to understand the impact on weight loss outcome at 6 months. Western Institutional Review Board granted exemption to the study as it is a retrospective analysis with no identifiable protected health information.

### Participants

Participants included paying customers of the Retrofit program who enrolled through an employer-sponsored program. Employers of participants had selected Retrofit as a subsidized weight management program for employees as part of their employer health benefits package. Customers were considered as eligible participants if they were at least 18 years of age; had a starting body mass index (BMI) of ≥ 25 kg/m^2^; had signed up for the program between September 27, 2011, and December 31, 2016; and had provided at least 1 weight measurement beyond baseline measurement. A participant was considered to have completed the program if he or she provided a weight measurement at the 6th month of his or her program. A total 1432 customers satisfied all inclusion criteria to be study participants, and 1045 of the participants completed the program. No customer was removed or eliminated from the population due to a lack of weight loss in the program.

### Program

The Retrofit weight loss program was designed with a 6-month weight loss phase with the option to continue into a maintenance program called Retrofit Next. The program (Multimedia Appendix 2) includes one-on-one expert coaching, unlimited coach interactions through electronic messaging, lifestyle patterns assessment, and personalized coaching content and plan. Expert coaches perform weekly reviews of participants’ plan and self-monitoring data to provide personalized feedback. Participants have access to an expert-moderated Web-based community and are encouraged to attend live weekly expert-led interactive Web-based classes regarding topics of exercise, nutrition, and mind-set. Digital tools, including a mobile app, Web-based dashboard, activity tracker, and Wi-Fi scale, are provided for tracking behaviors related to weight, food, mood, steps, and exercise.

As part of the Retrofit weight loss protocol, all participants are offered 7 one-on-one expert coaching sessions, including an initial 60-min session and 30-min follow-up sessions. Coaching sessions were conducted via Web-based video call or mobile phone. All coaching sessions include education around the Retrofit philosophy and weight loss guiding principles associated with nutrition, mind-set, exercise, and daily activities. In addition, each coaching session was used for coach-participant collaboration on current and desired health-related behaviors, goal setting to create individualized plans and strategies, and to come to an agreement on how the expert coach will hold the participant accountable to agreed-upon plans and strategies.

Participants were encouraged to weigh in, wear their activity tracker, log all food and beverages consumed, and communicate daily with their expert coach and in the Web-based community. Retrofit protocol required expert coaches to review a participant’s food and exercise logs, step data, weight data, and progress toward plan goals a minimum of 1 time per week to provide personalized feedback. If a participant initiated a coaching conversation, the expert coach was required to respond within 24 hours.

Retrofit expert coaches were employed professionals with a master’s or doctorate-level education in dietetics or nutritional sciences, exercise physiology, nursing, health education, counseling, or psychology. Expert coaches were certified in Retrofit’s weight loss protocol and have completed yearly recertification, if applicable.

### Measures

#### Weight

Participants were provided a Wi-Fi-enabled scale that securely transmitted weight data over the Internet to a Retrofit central data server. Participants’ weight data were collected through the use of the provided wireless scale (92% of recorded weights) or self-reported entry (8%). Self-reported entry was permissible if a participant had difficulty setting up his or her Wi-Fi scale. Baseline weight was considered as the first weight measurement received from the participant, which was designated as the recording for week 1. Percentage of baseline weight lost at 6 months was calculated and used as the primary outcome.

#### Expert Coaching Sessions

Participants were provided 7 one-on-one expert coaching sessions over the 6-month weight loss program. Percentage of coaching sessions attended at 6 months was calculated to quantify participant's engagement with their coach and used as one of the primary metrics to indicate coaching impact on participant outcome. A secondary metric was calculated to measure the total time a participant spent in coaching sessions.

#### Live Weekly Expert-Led Interactive Web-Based Classes

Participants were provided 26 weekly Web-based classes (1 class per week) where an expert coach conducted a live Web-based class on a predetermined topic. Percentage of classes attended at 6 months was calculated to quantify participants’ interest in gaining in-depth knowledge on a healthy lifestyle and weight management practices. A secondary metric was calculated to capture the total time a participant spent in weekly Web-based classes.

#### Coach-Participant Conversations

The total number of coach-participant conversations was calculated by counting all electronic messages including coach-initiated conversations, coach responses to participant-initiated conversations, and coach feedback on food or exercise logs. The total number of coach-participant conversation days was calculated by including all days when an expert coach sent at least 1 electronic message. The average conversation length per week was calculated by counting the average of total length of all electronic messages (in characters) sent in a week.

To evaluate the impact of food log feedback on weight loss outcome, we calculated several measures to capture coach-initiated electronic feedback messages that include evaluation and guidance in response to participants’ food logs. Total number of food log feedback counts all food log feedback provided by coach, which are defined as an expert coach comment written directly on a participant’s individual food log or weekly diary of food log entries entered through digital tools provided. The total number of food log feedback days was calculated by counting all days with at least 1 food log–related feedback from the expert coach. The average food log feedback length per week was measured by averaging the total length of all feedback messages (in characters) provided in a week. Similar to food log feedback, 3 measures for exercise log feedback were also calculated.

Finally, 3 measures were defined to measure participant engagement with coach. Similar to expert coach–initiated electronic message measures, the total number of participant-initiated electronic messages, the total number of participant-initiated electronic message days, and the average participant-initiated electronic message lengths per week were calculated.

### Statistical Analysis

All measures associated with coach-participant interactions involving expert coaching sessions, weekly Web-based classes, and electronic messaging and feedback were included in a multiple regression analysis to predict weight loss during the 6-month intervention. The least informative covariates were successively removed from the model in a stepwise elimination procedure based on the Akaike information criterion [25]. The regression model included only the main effects; interactions were beyond the scope of this analysis. In addition, this study focused on analyzing 3 statistically significant (*P*<.001) coaching interactions that were determined to be significant predictors in a weight loss model.

Data analyses were performed using R version 3.2.3 [26], which included dplyr 0.4.3, ggplot2 2.1.0, data.table 1.9.6, and leaps 2.9 packages. We also conducted *t* tests of equal variance on continuous variables at baseline and subsequent time points for 2 group comparisons. One-way analysis of variance (ANOVA) was utilized to determine mean differences for greater than 2 group comparisons. Subsequent Tukey tests were conducted to determine mean differences. Chi-square analyses were performed to determine differences among categorical variables when appropriate. For intent-to-treat (ITT) analyses, we used a last observation carried forward imputation approach. Alpha was set at .05 for all statistical tests to determine statistical significance.

## Results

The reported results are based on the retrospective analysis evaluating the effect of various coach-participant interactions during the Retrofit 6-month weight loss intervention using both the ITT (N=1432) and the completer (n=1045 participants) populations. First, a detailed analysis on different coach-participant interaction measures is provided to understand both coach and participant behavior over a 6-month weight loss intervention. Second, a multiple regression model is presented to capture interaction measures that significantly impact participant outcome at 6 months, and finally, an in-depth analysis is provided for the top 3 significant measures.

### Baseline Characteristics

Table 1 shows the demographic details at baseline for both ITT and completer populations. Although not clinically meaningful, the completers had higher average age compared with the overall population (45.73 vs 44.39, *P*=.001). Although there are differences in starting weight between completer and noncompleter groups, there are no differences in BMI at baseline between both populations. Furthermore, there are no differences in the male and female distribution among the ITT and completer groups (females: 61% vs 63%, *P*=.33).

### Weight Change at 6 Months

For ITT population, the average weight loss at 6 months was 5.14% (SE 0.12), and 44% of the participants lost 5% or more of their baseline weight (see Table 2). For completers, the average weight loss at 6 months was 6.15% (SE 0.17), and 54% of the participants lost 5% or more of their baseline weight. For both ITT and completers, there were no significant differences between males and females in terms of weight loss percentage or the percentage losing 5% or more weight at 6 months.

### Understanding Coach-Participant Interaction

The detailed quantitative analysis of the interaction between expert coach and participant is presented in Table 3. In general, completers had more interaction with coaches than the ITT population. The higher percentage of attendance or higher amount of interaction of the completers could be due to length of time actively participating in the weight loss program. Note that the average time in program for the noncompleters was about 3 months (mean 92.45 days, SE 2.20). In our analysis of the participant behavior below, we will focus on the ITT population.

Participants attended 75% of the one-on-one expert coaching sessions. Females attended higher percentage of coaching sessions than males (78.37% vs 70.72%, *P*<.001). Participants attended about 41% of the weekly Web-based classes. There is a gender difference observed in weekly Web-based class attendance as females attended significantly higher percentage of classes than males (51% vs 32%, *P*<.001). Consequently, females spent significantly higher amount of total time (638 min vs 405 min, *P*<.001) in classes learning about exercise, nutrition, and mind-set behaviors.

**Table 1:** Baseline demographics and outcome at 6 months.

Baseline demographics	Intent to treat (N=1432^a^), mean (SD)	Completers (n=1045^b^), mean (SD)	Noncompleters (n=387^c^), mean (SD)	*P* value^d^
Age, years	44.39 (10.31)	45.73 (10.10)	40.79 (10.00)	<.001
Starting weight, kg	104.76 (22.46)	103.95 (22.03)	106.94 (23.45)	.03
Starting body mass index, kg/m^2^	35.88 (6.56)	35.82 (6.46)	36.03 (6.81)	.62

^a^869 female, 563 male.

^b^655 female, 390 male.

^c^214 female, 173 male.

^d^Completer vs noncompleter.

**Table 2:** Weight loss outcomes at 6 months.

Population		Intent to treat			Completers		
		n (%)	Weight loss percentage, mean (SE)	Lost 5% or more of baseline weight, mean (SE)	n (%)	Weight loss percentage, mean (SE)	Lost 5% or more of baseline weight, mean (SE)
Overall		1432 (100.00)	5.14 (0.14)	44 (0.01)	1045 (100.00)	6.15 (0.17)	54 (0.02)
**Gender**							
	Female	869 (60.68)	5.19^a^ (0.14)	44^b^ (0.02)	655 (62.68)	6.00^c^ (0.17)	52^d^ (0.02)
	Male	563 (39.32)	5.06^a^ (0.14)	43^b^ (0.01)	390 (37.32)	6.40^c^ (0.18)	55^d^ (0.03)

^a^For ITT, the weight loss difference between female and male is not significant (*P*=.66).

^b^For ITT, the difference between percentage of female and male losing 5% is not significant (*P*=.73).

^c^For completers, the weight loss difference between female and male is not significant (*P*=.27).

^d^For completers, the difference between percentage of female and male losing 5% is not significant (*P*=.38).

**Table 3:** Coach-participant interaction measures at 6 months.

Interactions		Intent to treat (N=1432), mean (SE)	Completers (n=1045), mean (SE)
**Expert coaching sessions**			
	Percentage of coaching sessions attended	75.36 (0.72)	85.99 (0.61)
	Total time spent in coaching sessions, min	188.34 (1.61)	211.32 (1.41)
**Live weekly expert-led interactive Web-based classes**			
	Percentage of class attended	40.74 (0.83)	52.92 (0.92)
	Total time spent in class, min	546.70 (10.52)	663.31 (11.81)
**Coach-participant conversations**			
	Number of coach messages	158.91 (2.36)	180.00 (2.82)
	Number of coach message days	75.16 (0.65)	82.36 (0.65)
	Coach message length/week, characters	1458.34 (13.79)	1434.34 (20.94)
	Number of food log feedback	74.91 (1.82)	89.27 (2.26)
	Number of food log feedback days	31.89 (0.50)	37.01 (0.56)
	Food log feedback length/week	409.29 (6.69)	410.05 (7.56)
	Number of exercise log feedback	16.69 (0.32)	19.21 (0.38)
	Number of exercise log feedback days	12.89 (0.23)	14.6 (0.26)
	Exercise log feedback length/week	187.56 (3.89)	180.42 (4.22)
	Number of participant messages	48.89 (1.27)	58.54 (1.60)
	Number of participant message days	29.02 (0.64)	34.67 (0.77)
	Participant message length/week, characters	399.29 (9.71)	433.12 (12.37)

**Table 4:** Multiple regression models identifying predictors of weight loss at 6 months. Multiple regression model summary: R2=.158; adjusted R2=.152, *P*<.001.

Models	Coefficients		
	β ( SE)	*t* (degrees of freedom=997)	*P* value
Percentage of coaching sessions attendance	−1.05 (0.21)	−4.90	<.001
Percentage of weekly class attendance	−.76 (0.21)	−3.66	<.001
Number of food log feedback days	−.92 (0.26)	−3.50	<.001
Total number of coach message days	.89 (0.31)	2.83	.005
Coach message length per week	.54 (0.17)	3.14	.002
Number of participant messages	.95 (0.56)	1.68	.09
Number of participant message days	−1.56 (0.60)	−2.59	.01

Furthermore, coach-participant conversations were reviewed to assess the amount of interactions over the 6-month program. On an average, an expert coach reached out to his or her participant with responses, food/exercise log feedback, or general weight management guidelines approximately 75 days within the 6-month program (about 3 times a week). In general, participants who were more engaged in the program by initiating more conversations or logged more food/exercise logs received higher amount of communication from coaches. In addition, females received higher number of coach messages than males (170.24 vs 141.42, *P*<.001).

As reported in Table 3, almost half of the coach conversations were food log feedback (74.91 out of 158.91 messages). Females received significantly higher number of food log feedback than males (81.96 vs 64.04, *P*<.001). As females logged a higher number of food logs capturing their daily food intakes, coaches provided a higher amount of feedback. Participants either initiated conversation or responded to coach messages at least once a week (33.28 days) on average. Females sent higher number of messages than males (56.41 vs 37.28, *P*<.001 *)*.

### Multiple Regression Model for Coach-Participant Interactions

A multiple regression model was built to predict weight change at 6 months by including all interaction measures related to coaching sessions, weekly Web-based classes, and coach-participant conversations. In the backward stepwise elimination multiple regression analysis, the final model (*R*^2^=.158, *P*<.001) included 7 coach-participant interaction measures, in which 6 of the measures were identified as statistically significant predictors: percentage of coaching sessions completed (β=−1.05, SE 0.21, *P*<.001), percentage of class attended (β=−.76, SE 0.21, *P*<.001), number of food log feedback days (β=−.92, SE 0.26, *P*<.001), total number of coach message days (β=.89, SE 0.31, *P*=.005), coach message length per week (β=.54, SE 0.17, *P*=.002), and number of participant message days (β=−1.56, SE 0.60, *P*=.01). The best regression model containing 7 coach-participant interaction measures is reported in Table 4.

### Significant Weight Loss Predictors: In-Depth Analysis

This section focuses on analyzing 3 of the predictors from the final regression model in Table 4, which have *P*<.001: percentage of coaching sessions completed, percentage of weekly classes completed, and number of food log feedback days. These analyses focus on quantifying different levels of coaching interaction and corresponding weight loss at 6 months to characterize the association with outcome. In addition, average coaching interactions were calculated for participants with different levels of weight loss at 6 months: lost ≥10% (264/1432, 18.44%), lost 5% to 10% (366/1432, 25.56%), and lost <5% (802/1432, 56.01%).

#### Expert Coaching Sessions

**Figure 1:**
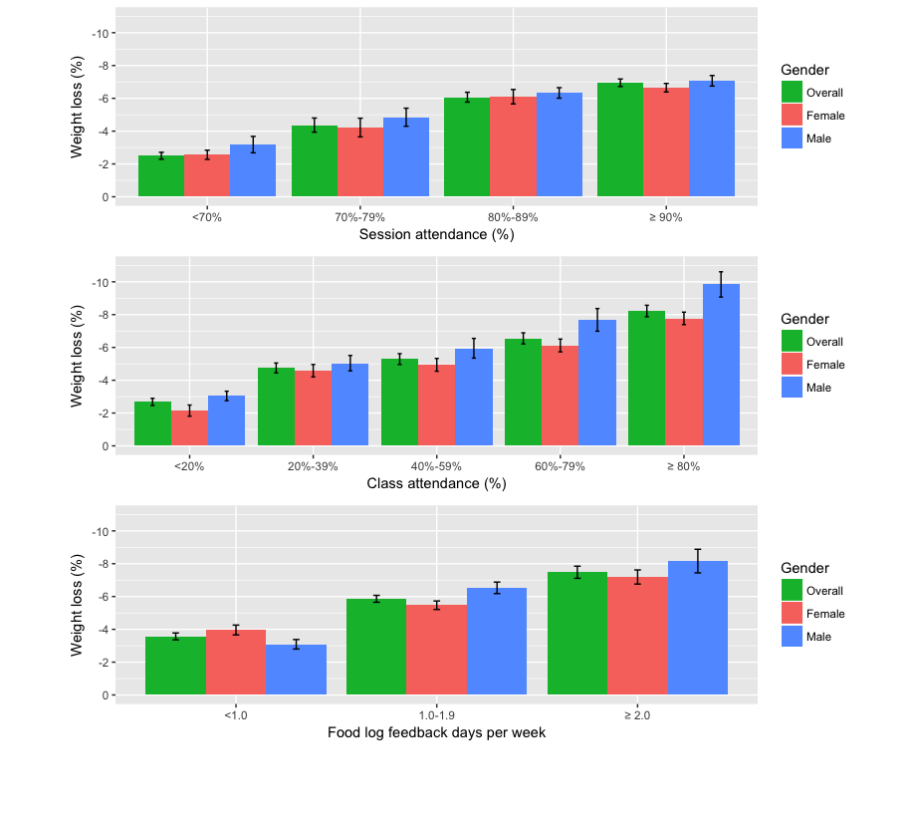
Weight loss outcomes for different levels of coach-participant interaction.

On the basis of the percentage of coaching session attendance data from the 6-month program, a higher percentage of coaching session attendance is significantly associated with a higher level of weight loss at 6 months. As shown in Figure 1, clinically significant weight loss (5%) was associated with at least 80% of coaching session attendance. The results of one-way ANOVA showed a significant difference of mean weight loss between different weigh-in levels (*P*<.001). A subsequent Tukey test confirmed the significant differences among the 80% to 90% and ≥90% attendance levels with the lower 2 levels (*P*<.001). Similar ANOVA tests were performed on male and female participants separately, and a significant difference in mean weight loss between different attendance levels was found (male: *P*<.001; female: *P*<.001).

**Figure 2:**
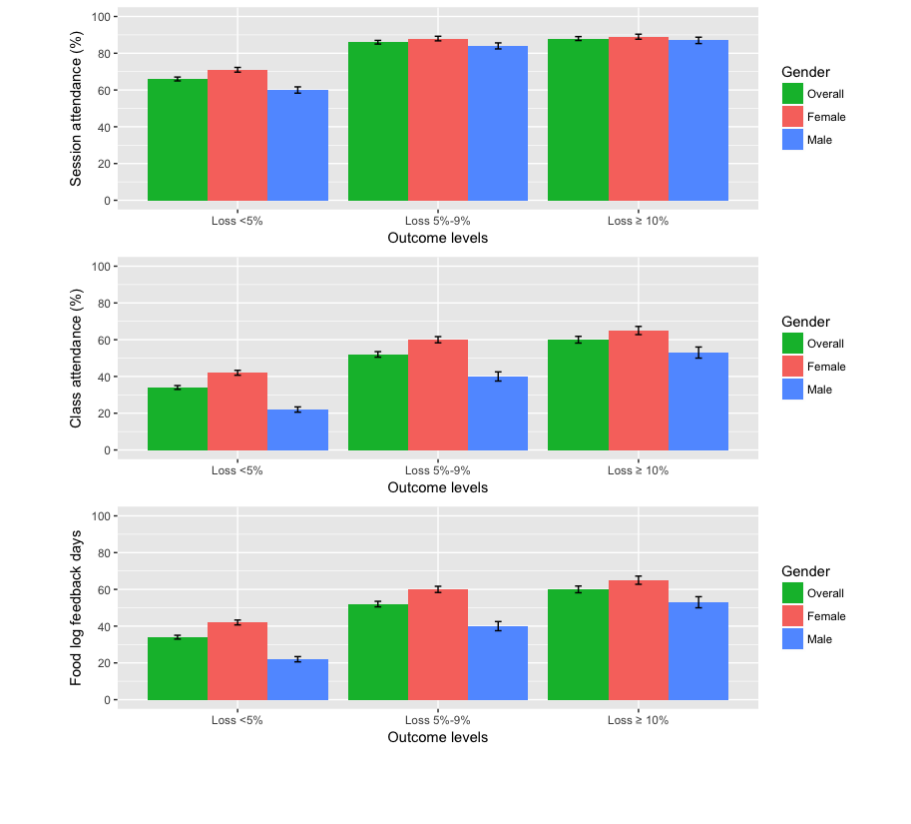
Interaction levels of participants with different levels of outcome.

Further analysis of coaching session attendance of participants with different levels of weight loss showed that a higher coaching session attendance was significantly associated with groups with higher levels of weight loss. Figure 2 shows a clear difference in coaching session attendance between loss <5% group and other 2 groups (*P*<.001). Both male and female participants separately showed a similar significant difference in coaching session attendance.

#### Live Weekly Expert-Led Interactive Web-Based Classes

As reported in Figure 1, the association between the percentage of weekly Web-based class attendance and weight loss at 6 months is linear where higher level of weight loss is significantly associated with higher percentage of class attendance. Clinically significant weight loss is associated with at least 60% of class attendance for overall and both male and females separately. One way ANOVA and a subsequent Tukey test confirmed significant mean differences in weight loss among 60% to 80% and ≥80% groups with the remaining levels of class attendance (*P*<.001). The analysis of percentage of class attendance of participants with different levels of weight loss showed that a higher class attendance was significantly associated with groups with higher levels of weight loss. Male and female participants separately showed similar significant differences in mean percentage of class attendance between different outcome levels (male: *P*<.001; female: *P*<.001).

#### Food Log Feedback Days

A higher number of food log feedback days per week is significantly associated with higher level of weight loss at 6 months. One way ANOVA test showed a significant mean difference in weight loss between difference in food log feedback levels (*P*<.001). A subsequent Tukey test confirmed significant mean differences between all levels of food log feedback days. Further analysis of food log feedback days of participants with different levels of weight loss showed that higher counts of food log feedback days were significantly associated with groups with higher levels of weight loss (*P*<.001).

## Discussion

### Principal Findings

The results provide strong support for expert coaches in weight management programs. Participants had greater weight loss with a higher attendance of expert coaching sessions and live weekly expert-led interactive Web-based classes, as well as higher engagement with an expert coach through food log feedback. Completers also had greater interaction and attendance than ITT. In a multiple regression analysis, 6 of the 7 interaction measures were identified as statistically significant predictors of weight loss. In addition, an in-depth analysis of the top 3 significant predictors quantified the impact of coaching sessions completed, weekly Web-based class attendance, and days of receiving food log feedback on varying levels of weight loss. Overall, expert coaches were found to have a high impact on weight management.

Expert coaches provide guidance and accountability to increase participant engagement and weight loss success, which is supported by previous studies, including website, email, and/or mobile phone apps, as well as interventions using only phone calls for coaching [7-9,27]. However, the participant must be actively engaged in the program to receive benefit of the interactions. Quantifying the minimum and maximum level of engagement for significant weight loss can drive best practices for weight management expert coaches.

Although consistent self-monitoring is shown to have a predictive value for weight loss, the challenge is maintaining consistency among participants [20]. Findings support previous studies that personalized feedback and communication from expert coaches can produce greater engagement in self-monitoring activities when compared with tech-based interventions for self-monitoring without expert feedback [23,28,29]. We found that expert coaching sessions, live weekly expert-led classes, and food log feedback specifically increased interaction and have predictive weight loss values. On the basis of these results, it may be important to promote these coach-participant interactions together in an intervention or weight loss program.

### Significant Predictors of Weight Loss

#### Expert Coaching Session Attendance

The percentage of coaching sessions completed was identified as a significant predictor of weight loss (*P*<.001). Attending 80% of the offered coaching sessions is associated with clinically significant weight loss of 5% or more. Others have shown that weekly to monthly coaching sessions are linked with 5% to 10% weight loss, improved adherence to health strategies, and decreased risk factors over a 6- to 12-month intervention [14-17]. Overall, female participants attended more coaching sessions than male participants, yet no significant difference was found in weight loss outcomes. Similar observations were reported in prior studies where male participants did not utilize expert coaches as frequently as female participants [12,13,15].

#### Live Weekly Expert-Led Interactive Web-based Classes

The percentage of weekly Web-based classes completed was identified as a significant predictor of weight loss (*P*<.001). Clinically significant weight loss of 5% is associated with at least 60% class attendance overall and between male and females separately. However, class attendance above 60% was associated with greater weight loss among all groups. Higher class attendance was linked to participants achieving 5% to 10% and >10% weight loss, yet male and female differed in class percentage attendance associated with levels of outcome. Males had a significantly lower attendance rate than females, which is historically common in weight loss interventions [12,13,15].

#### Food Log Feedback Days

The number of food log feedback days per week was identified as a significant predictor of weight loss (*P*<.001). Participants receiving food log feedback 1 to 2 days per week and ≥2 days per week were associated with clinically significant weight loss of 5% or greater. Additionally, participants in the 5% to 10% and >10% weight loss levels received more food log feedback days than those in the <5% weight loss level regardless of group. Food log feedback is directly dependent upon the participant’s engagement in providing food logs for an expert coach to review. Females received a greater amount of feedback due to logging a higher number of food logs than males, which has been reported in earlier studies [20]. However, this finding is linked to the understanding that personalized feedback increases engagement and weight loss outcomes [22-24].

### Strengths and Limitations

This study has several strengths, including the reporting of real-world weight loss outcomes and a focused analysis into expert coaches’ role in a weight management program to determine which coach-participant interactions have a significant impact on participant success. Participants were existing participants of Retrofit and not recruited or incentivized to participate in the study. All participants who met the starting BMI, age, and weight criteria and provided at least 1 weight measurement beyond baseline were included as participants. No participant was removed from the population because of lack of success on the program, which is an uncommon research practice in the weight management field [30]. This study provides further insight on best practices of expert coaches in weight management interventions and programs. In addition, with the high population of male participants, gender comparisons were reported to create a greater understanding of interaction between male participants and coaches.

The study has limitations, which include the retrospective analysis study design that does not provide any causal inferences based on the critical observations. Coach-participant interaction was measured from a quantitative point of view. Also, the use of a real-world population does not reveal whether a participant was actively using any other weight management program outside of the Retrofit program components.

### Future Research

Retrofit encourages all commercial weight loss programs to publish real-world research to enhance the understanding of coach-participant interactions in weight loss programs. Reporting real-world data in relation to expert coaches allows commercial weight loss program to structure protocols for participant engagement and adherence to weight loss strategies. By fine-tuning interactions and by understanding how expert coaches are most effective, commercial weight loss programs will increase capability in overcoming the obesity crisis.

Recommended future research includes an analysis of specific strategies used by expert coaches and their impact on weight loss outcomes, as well as a qualitative analysis of the interactions between a coach and a participant, which may provide more insight into an expert coach’s impact on participants. With the continued observation in this study and previous studies that male participants are less engaged than females, an analysis of strategies to increase male engagement and to understand whether increased engagement improves male weight loss outcomes is recommended. Additionally, further research is needed to analyze coaching impact on participants’ self-monitoring behaviors to determine association between coach-participant interaction and the level of self-monitoring behaviors. Finally, expert coaches’ impact beyond an initial 6-month intervention and the impact of each predictor of weight loss on weight maintenance would be a valuable future research study.

### Conclusions

In conclusion, participants on the Retrofit weight loss program lost on average 5.14% (SE 0.14), and participants who completed the program lost on average 6.15% (SE 0.17) in 6 months. Over half of completers (54%) and 44% of all participants lost 5% or more of their baseline weight. Coach-participant interactions that include one-on-one expert coaching session attendance, live weekly expert-led interactive Web-based class attendance, and food log feedback days per week were shown to be significant predictors of weight change at 6 months. Specifically, attending 80% or more of offered expert coaching sessions, attending 60% or more of offered weekly Web-based classes, and receiving food log feedback one or more days per week from an expert coach increased participants’ weight loss success.

## Appendix

Multimedia Appendix 1 Retrofit logo.

Multimedia Appendix 2 Features of the Retrofit Weight Loss program.

## Abbreviations

ANOVA: analysis of variance
BMI: body mass index
ITT: intent to treat

## References

[1] World Health Organization.

[2] Waters H, DeVol R Milken Institute.

[3] Gates DM, Succop P, Brehm BJ, Gillespie GL, Sommers BD (2008). Obesity and presenteeism: the impact of body mass index on workplace productivity. J Occup Environ Med.

[4] Van Nuys K, Globe D, Ng-Mak D, Cheung H, Sullivan J, Goldman D (2014). The association between employee obesity and employer costs: evidence from a panel of U.S. employers. Am J Health Promot.

[5] National Center for Health Statistics.

[6] The Henry J. Kaiser Family Foundation.

[7] van Wier MF, Ariëns GA, Dekkers JC, Hendriksen IJ, Smid T, van Mechelen W (2009). Phone and e-mail counselling are effective for weight management in an overweight working population: a randomized controlled trial. BMC Public Health.

[8] Vale MJ, Jelinek MV, Best JD, Santamaria JD (2002). Coaching patients with coronary heart disease to achieve the target cholesterol: a method to bridge the gap between evidence-based medicine and the “real world”--randomized controlled trial. J Clin Epidemiol.

[9] Vale MJ, Jelinek MV, Best JD, Dart AM, Grigg LE, Hare DL, Ho BP, Newman RW, McNeil JJ, COACH Study Group (2003). Coaching patients On Achieving Cardiovascular Health (COACH): a multicenter randomized trial in patients with coronary heart disease. Arch Intern Med.

[10] Heinen L, Darling H (2009). Addressing obesity in the workplace: the role of employers. Milbank Q.

[11] Xiao L, Yank V, Wilson SR, Lavori PW, Ma J (2013). Two-year weight-loss maintenance in primary care-based Diabetes Prevention Program lifestyle interventions. Nutr Diabetes.

[12] Gabriele JM, Carpenter BD, Tate DF, Fisher EB (2011). Directive and nondirective e-coach support for weight loss in overweight adults. Ann Behav Med.

[13] Brace AM, Padilla HM, DeJoy DM, Wilson MG, Vandenberg RJ, Davis M (2015). Applying RE-AIM to the evaluation of FUEL Your Life : a worksite translation of DPP. Health Promot Pract.

[14] Leahey TM, Wing RR (2013). A randomized controlled pilot study testing three types of health coaches for obesity treatment: professional, peer, and mentor. Obesity (Silver Spring).

[15] Kirkman MS, Weinberger M, Landsman PB, Samsa GP, Shortliffe EA, Simel DL, Feussner JR (1994). A telephone-delivered intervention for patients with NIDDM. Effect on coronary risk factors. Diabetes Care.

[16] Tate DF, Jackvony EH, Wing RR (2003). Effects of Internet behavioral counseling on weight loss in adults at risk for type 2 diabetes: a randomized trial. J Am Med Assoc.

[17] Eakin EG, Lawler SP, Vandelanotte C, Owen N (2007). Telephone interventions for physical activity and dietary behavior change: a systematic review. Am J Prev Med.

[18] Tate DF, Wing RR, Winett RA (2001). Using Internet technology to deliver a behavioral weight loss program. J Am Med Assoc.

[19] Carney D, Schultz S, Lim J, Walters W (2015). Successful medical weight loss in a community setting. J Obes Weight Loss Ther.

[20] Painter SL, Ahmed R, Hill JO, Kushner RF, Lindquist R, Brunning S, Margulies A (2017). What matters in weight loss? An in-depth analysis of self-monitoring. J Med Internet Res.

[21] Hutchesson MJ, Tan CY, Morgan P, Callister R, Collins C (2016). Enhancement of self-monitoring in a web-based weight loss program by extra individualized feedback and reminders: randomized trial. J Med Internet Res.

[22] Castro CM, Pruitt LA, Buman MP, King AC (2011). Physical activity program delivery by professionals versus volunteers: the TEAM randomized trial. Health Psychol.

[23] Tate DF, Jackvony EH, Wing RR (2006). A randomized trial comparing human e-mail counseling, computer-automated tailored counseling, and no counseling in an Internet weight loss program. Arch Intern Med.

[24] Sherrington A, Newham JJ, Bell R, Adamson A, McColl E, Araujo-Soares V (2016). Systematic review and meta-analysis of internet-delivered interventions providing personalized feedback for weight loss in overweight and obese adults. Obes Rev.

[25] Akaike H (1974). Bayes.acs.unt.edu.

[26] R-project.

[27] Tao M, Rangarajan K, Paustian ML, Wasilevich EA, El Reda DK (2014). Dialing in: effect of telephonic wellness coaching on weight loss. Am J Manag Care.

[28] Gold BC, Burke S, Pintauro S, Buzzell P, Harvey-Berino J (2007). Weight loss on the web: a pilot study comparing a structured behavioral intervention to a commercial program. Obesity (Silver Spring).

[29] Turk MW, Elci OU, Wang J, Sereika SM, Ewing LJ, Acharya SD, Glanz K, Burke LE (2013). Self-monitoring as a mediator of weight loss in the SMART randomized clinical trial. Int J Behav Med.

[30] Gudzune KA, Bleich SN, Clark JM (2015). Efficacy of commercial weight-loss programs. Ann Intern Med.

