# Supplementary figures and images for: Expert Coaching in Weight Loss: Retrospective Analysis
Source: J Med Internet Res. 2018 Mar 13;20(3):e92. doi: 10.2196/jmir.9738 (PMC5871741; doi:10.2196/jmir.9738)

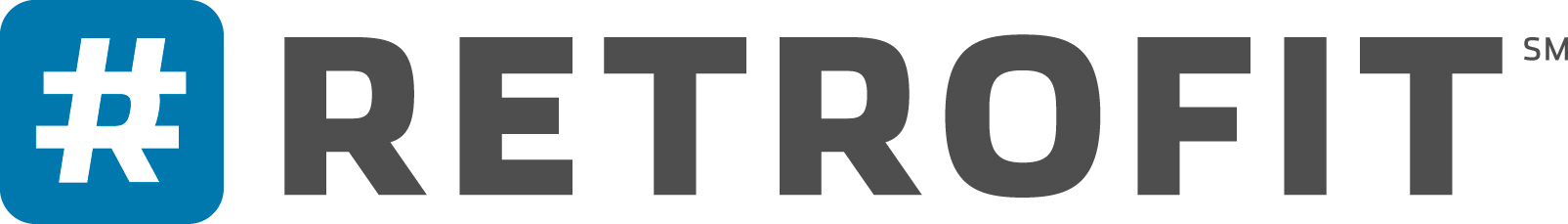

Supplement: Multimedia Appendix 1 [file jmir_v20i3e92_app1.jpg]

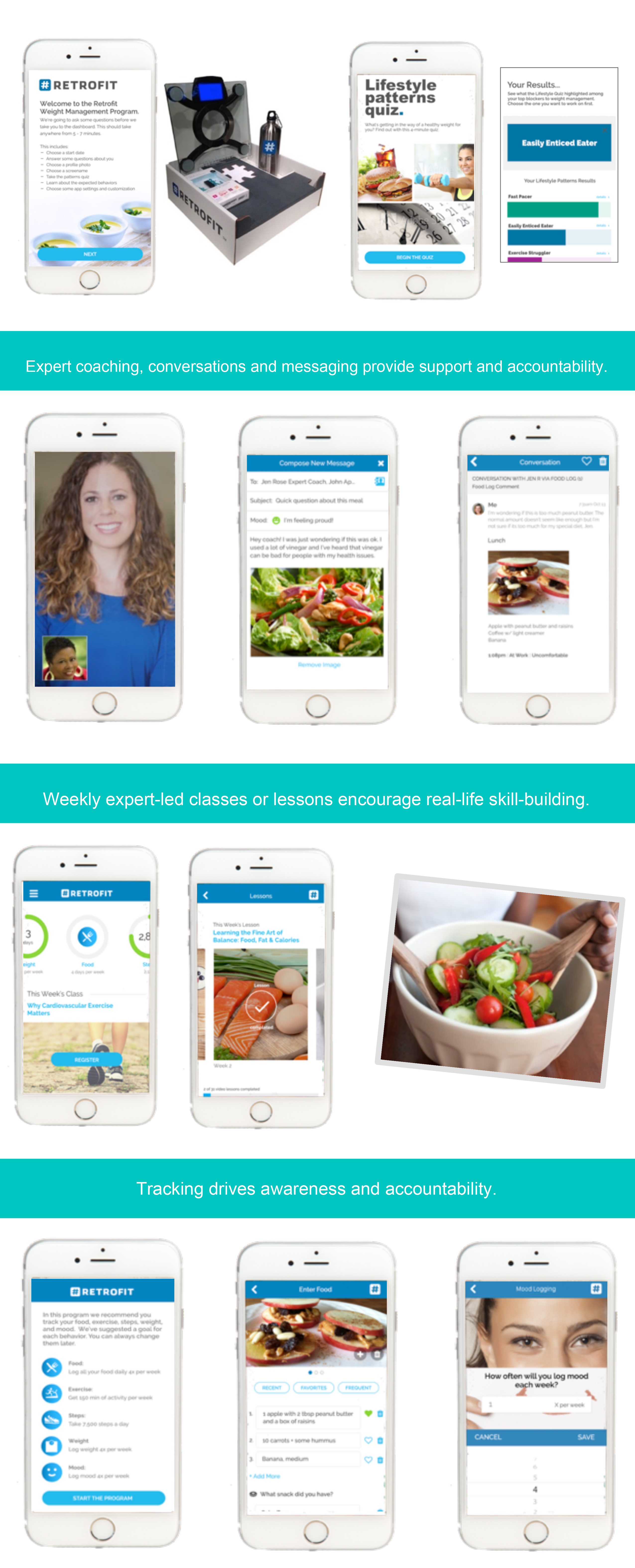

Supplement: Multimedia Appendix 2 [file jmir_v20i3e92_app2.jpg]
